# A case report: anesthetic management of a patient with unexpected difficult airway due to an asymptomatic giant epiglottic cyst

**DOI:** 10.1186/s12871-026-04181-4

**Published:** 2026-08-13

**Authors:** Xiaojun Wang, Lin Li, Zhilin Liu, Meilin Ni, Cong Wei, Ting Yi, Wuwei Zheng

**Affiliations:** Department of Anesthesiology, The Affiliated Yangming Hospital of Ningbo University (Yuyao People’s Hospital), Yuyao, Zhejiang Province P. R. China

**Keywords:** Epiglottic cyst, Difficult airway, Intubation, Anaesthesia evaluation, Video laryngoscopy, Case report

## Abstract

**Background:**

Giant asymptomatic epiglottic cysts are rare, benign lesions that can cause life-threatening airway obstructions during general anaesthesia induction.

**Objective:**

To describe the clinical recognition, perioperative evaluation, and successful anaesthetic management of an unexpected difficult airway caused by an undiagnosed giant epiglottic cyst and to share the clinical experience.

**Case presentation:**

A 60-year-old woman presented for elective laparoscopic cholecystectomy. After induction, video laryngoscopy revealed a giant cyst obscuring the view of the glottis. The airway was successfully secured using a two-person cooperative manoeuvre, with mask ventilation remaining effective throughout.

**Conclusion:**

Comprehensive preoperative assessment is critical; however, asymptomatic giant epiglottic cysts present a hidden airway risk. Timely video laryngoscopy and adequate support ensure patient safety.

**Supplementary Information:**

The online version contains supplementary material available at https://doi.org/10.1186/s12871-026-04181-4.

## Background

Epiglottic cysts are common benign hypopharyngeal lesions [1]. They are primarily caused by obstruction of the mucous gland ducts in the epiglottic mucosa or by congenital abnormalities [2]. While small cysts are generally asymptomatic and discovered incidentally during routine examinations, clinical manifestations may emerge once the cyst enlarges. When the volume of the cyst increases to a certain extent, symptoms such as sore throat, dysphagia, or dyspnoea may occur [3, 4]. In severe cases, complete airway obstruction may develop, constituting a life-threatening anaesthetic emergency. However, giant epiglottic cysts that cause an unexpectedly difficult airway may present no preoperative warning signs. Improper anaesthesia induction management may cause cyst displacement and airway obstruction, leading to fatal complications, including asphyxia and cardiac arrest [5]. Although numerous reports have detailed the anaesthetic management of symptomatic epiglottic cysts, asymptomatic giant cysts presenting with isolated intubation difficulty (without ventilation impairment) remain extremely rare [4, 6].

## Case presentation

### Preoperative clinical status

A 60-year-old female (height, 1.55 m; weight, 57.1 kg) presented with recurrent epigastric pain for 1 year, with acute exacerbation during the 3 days prior to admission. Imaging confirmed cholecystitis, multiple gallstones, and biliary sludge, and elective laparoscopic cholecystectomy was scheduled. The patient had a 10-year history of grade 2 hypertension (moderate risk), which was treated with amlodipine besylate (5 mg once daily) and valsartan/hydrochlorothiazide (once daily). She also had a 1-year history of middle cerebral artery occlusion. Aspirin (100 mg once daily) had been discontinued 1 week before surgery, whereas atorvastatin calcium (20 mg once daily) was continued.

### Preoperative anaesthesia assessment

The patient was classified as American Society of Anesthesiologists (ASA) II or Mallampati II. She reported occasional snoring and a nonspecific pharyngeal foreign body sensation. This sensation was attributed to pharyngitis and was not evaluated further. She reported no other airway symptoms, such as sore throat, dysphagia, or dyspnoea. Neck movement and mouth opening were normal. Laboratory tests revealed elevated liver enzyme levels (alanine aminotransferase, 349 U/L; aspartate aminotransferase, 226 U/L) and bilirubin levels (total bilirubin, 70.1 µmol/L; direct bilirubin, 34.8 µmol/L; and indirect bilirubin, 35.3 µmol/L), consistent with cholecystitis. Chest CT was unremarkable, and preoperative laryngoscopy was not performed. No clear difficult airway risk factors were identified; therefore, routine tracheal intubation under general anaesthesia was planned.

### Preoperative preparation

Routine anaesthetic equipment, including an anaesthesia machine, masks, laryngoscopes, 7.0 mm tracheal tubes, stylets, and suction devices, was prepared, along with a video laryngoscope. Intraoperative monitoring included electrocardiography, non-invasive blood pressure measurement, heart rate, SpO_2_, and ETCO_2_.

### Airway treatment during anaesthesia induction

**Fig. 1:**
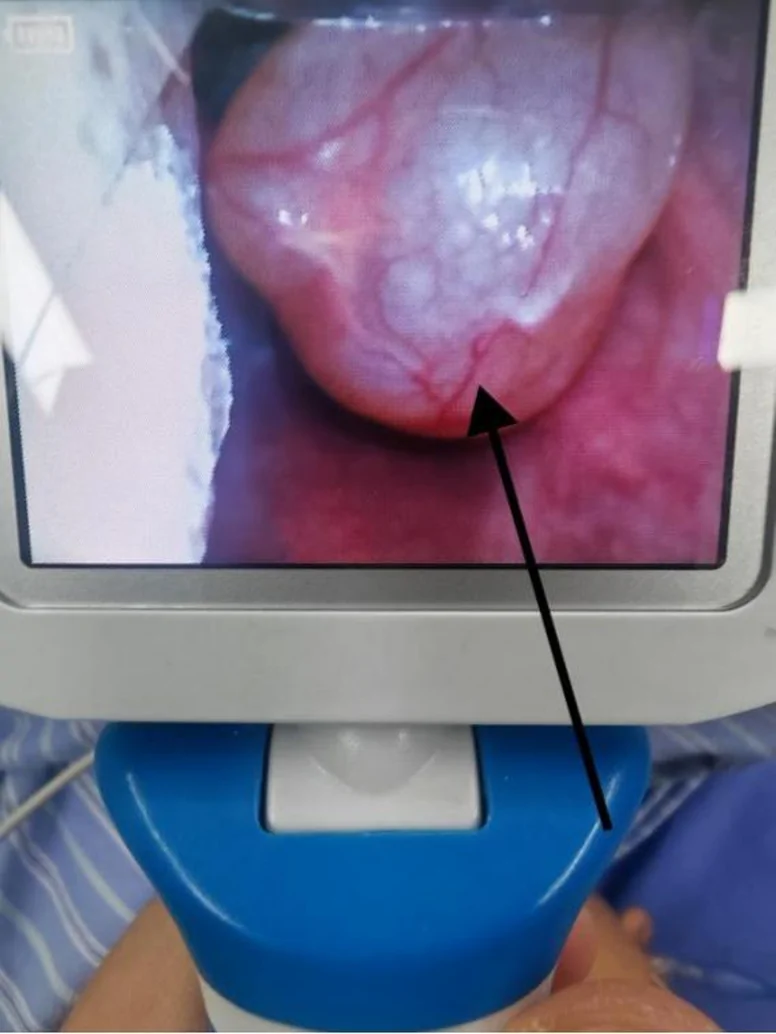
Video laryngoscopic view showing a giant cyst on the lingual surface of the epiglottis that completely obscured the glottis

On the day of surgery, the patient was transferred to the operating room. Two anaesthesiologists were present. Standard monitoring was used, and the following baseline vital signs were recorded: respiratory rate, 18 breaths/min; heart rate, 80 bpm; blood pressure, 150/89 mmHg; and SpO_2_, 100%. After 3 min of standard preoxygenation, anaesthesia was induced with intravenous remimazolam (10 mg), selected for its haemodynamic stability in this hypertensive patient, sufentanil (25 µg), cisatracurium (10 mg), and propofol (80 mg). Video laryngoscopy performed by the junior anaesthesiologist revealed a giant epiglottic cyst completely obscuring the glottis (Fig. 1). Recognizing the high risks of life-threatening cyst rupture, aspiration, and haemorrhage, the junior anaesthesiologist immediately aborted the intubation attempt. The team members’ roles were immediately reallocated: the senior anaesthesiologist took over airway management, while the junior anaesthesiologist continued to manage medication administration and monitoring. Concurrently, the circulating nurse activated the hospital’s difficult-airway protocol and called an otolaryngologist to the operating room for emergency tracheotomy standby. During the next attempt, the junior anaesthesiologist applied external laryngeal manipulation under the guidance of the senior anaesthesiologist. The senior anaesthesiologist gently elevated the cyst using the tip of the 7.0 mm endotracheal tube, exposing the posterior glottic commissure, and then advanced the tube smoothly into the trachea. The entire airway establishment process lasted approximately 10 min. After the single failed intubation attempt by the junior anaesthesiologist and before the senior anaesthesiologist performed the laryngoscopy, gentle low-pressure intermittent mask ventilation was provided. This interval ventilation maintained sufficient systemic oxygenation throughout the entire procedure, and all vital signs remained stable (heart rate, 70 bpm; blood pressure, 140/80 mmHg; SpO_2_ 100%; and ETCO_2_, 37 mmHg). Retrospective review of the preoperative chest CT images, which do not routinely cover the epiglottic region, revealed no evidence of the lesion. Laparoscopic cholecystectomy was completed uneventfully. The patient underwent awake extubation in the operating room and was transferred to the post-anaesthesia care unit (PACU) with stable vital signs. She was discharged home on postoperative day 2 without complications.

## Anaesthetic airway management plan for epiglottic cyst resection

Following the diagnosis of an asymptomatic giant epiglottic cyst after cholecystectomy, the patient was scheduled for elective cyst resection 1 month later.

**Fig. 2:**
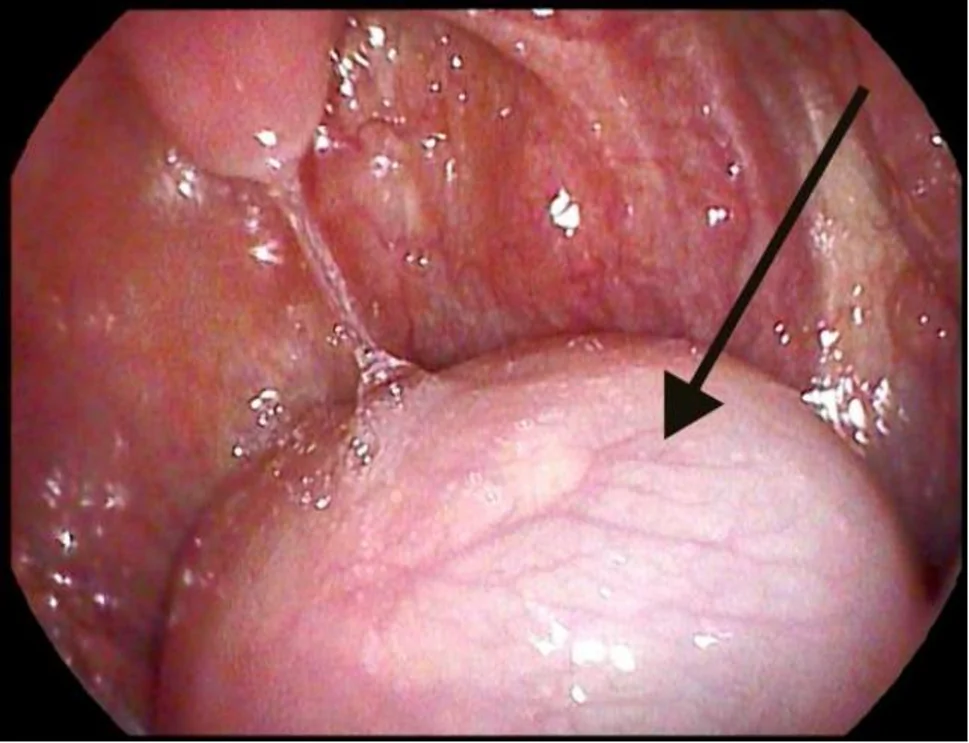
Preoperative flexible laryngoscopy: view of the epiglottic cyst

**Fig. 3:**
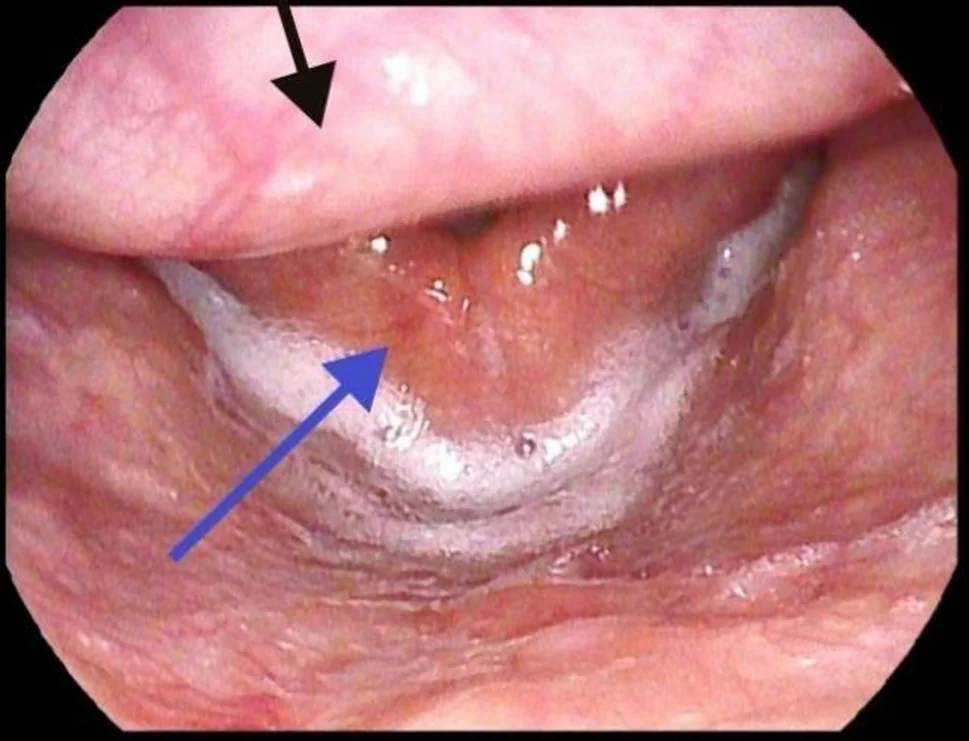
Preoperative flexible laryngoscopy: blue arrow, normal epiglottis; black arrow, cyst at the base of the epiglottic vallecula of the tongue

**Fig. 4:**
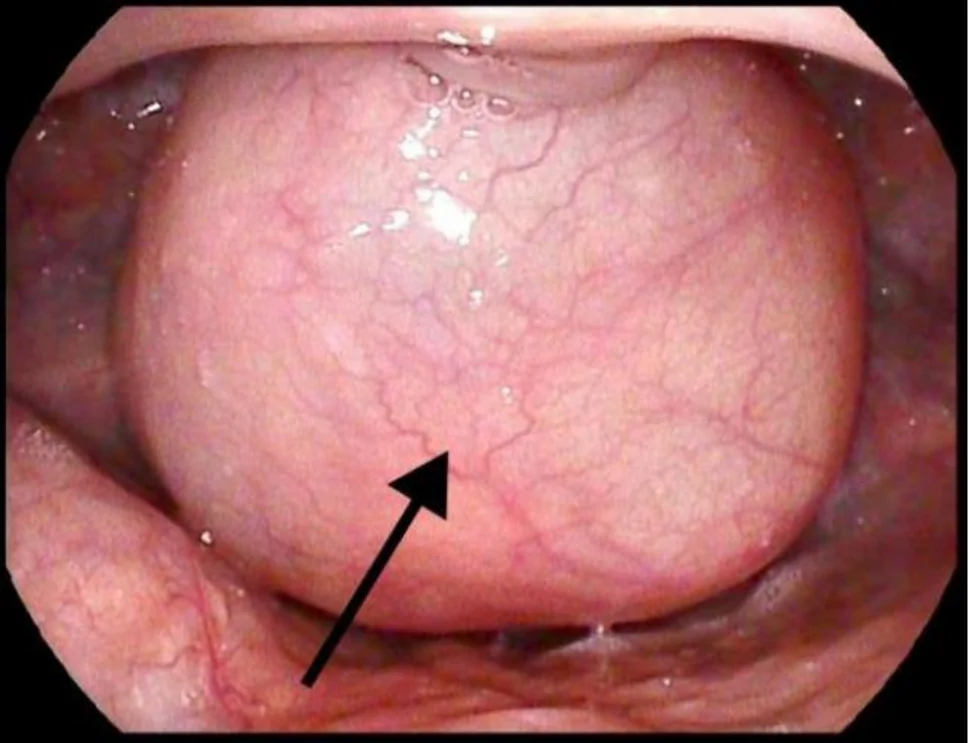
Preoperative flexible laryngoscopy showing a 2.0 × 2.0 cm cyst with a pedicle on the lingual surface of the epiglottis

Given the anticipated difficult airway, a standardized management plan was formulated preoperatively: ① Two anaesthesiologists were assigned to the procedure, at least one of whom was a deputy chief physician or higher. An otolaryngologist remained on standby throughout; ②Video laryngoscopy was designated the first-line intubation tool, with a fibreoptic bronchoscope prepared as a secondary option. Additional rescue supplies, including a 7.0 mm reinforced tube, an emergency tracheotomy kit and a cricothyroid puncture needle, were available as stepwise (non-invasive to invasive) backups; ③ Preoperative flexible laryngoscopy was performed to evaluate the location and anatomical characteristics of the cyst (Figs. 2, 3 and 4); ④ Anaesthesia induction involved standard intravenous induction, with continuous SpO_2_ monitoring maintained throughout; ⑤ Intraoperative monitoring: Airway pressure and ETCO_2_ were continuously monitored, and all manipulations were performed gently to avoid mucosal injury and oedema; and ⑥ Postoperative extubation: Extubation was performed only after the patient achieved stable spontaneous respiration.

**Fig. 5:**
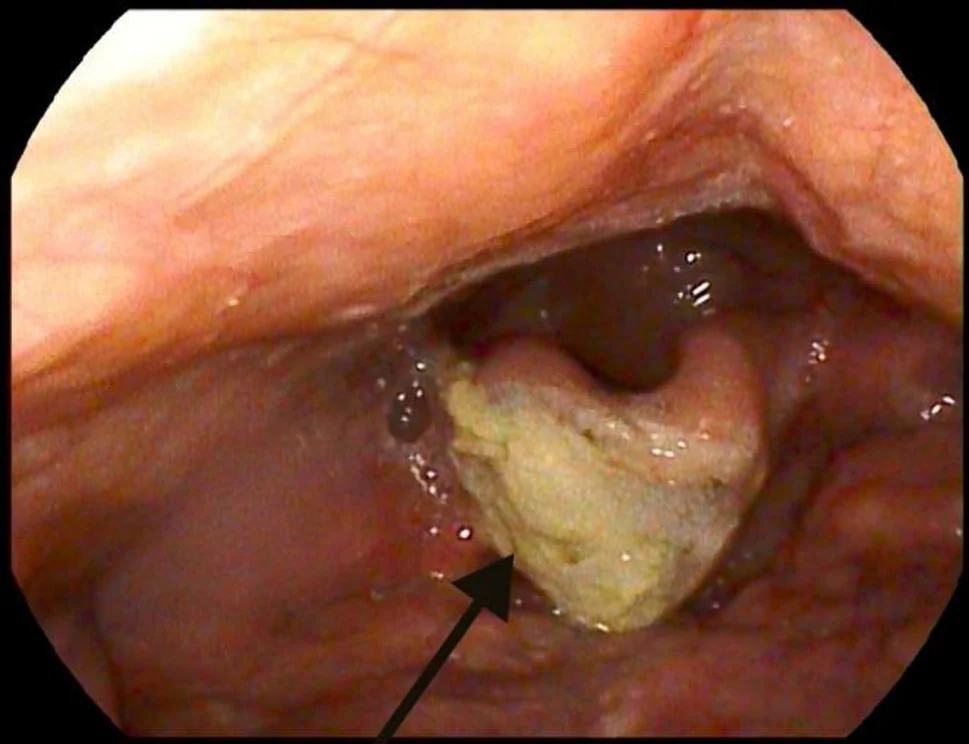
Two-week post-operative flexible laryngoscopic view showing the surgical site

Two weeks postoperatively, follow-up laryngoscopy revealed pale yellow crusting at the epiglottic surgical site, with no congestion, oedema, remnant, or recurrence (Fig. 5).

## Discussion

Epiglottic cysts primarily originate from obstruction of mucous gland ducts, grow slowly, and often cause no obvious symptoms in their early stages [7]. When located on the lingual surface of the epiglottis, they can occupy the supraglottic space and distort the local anatomy, leading to unexpectedly difficult intubation during anaesthesia induction [8, 9]. This case involved a typical asymptomatic giant epiglottic cyst that caused an unexpectedly difficult airway. The patient had no obvious airway symptoms, chest CT was unremarkable, and the Mallampati classification was Grade II. There were no clear risk factors for a difficult airway. Thus, the occult lesion was not detected during the preoperative evaluation.

Following anaesthesia induction, muscle relaxants cause upper airway muscle relaxation and cyst displacement, resulting in difficult glottis visualization. Blind intubation may lead to cyst rupture, bleeding, or aspiration. Fortunately, mask ventilation remained effective throughout. We speculate that the intact and tough cyst wall maintained a small ventilation space; however, this remains a clinical inference that was not further verified. The anaesthetic management in this case reflected a timely emergency response, appropriate device selection, standardized operation, and the importance of multidisciplinary collaboration [10].

Unlike most single-operator intubations reported in the literature, a two-person cooperative manoeuvre was adopted to secure the airway safely. Once the cyst was recognized, blind intubation attempts were abandoned immediately to avoid complications. Moreover, the difficult airway protocol was activated promptly [11], and an otolaryngologist was consulted for emergency standby [12]. Notably, the patient’s SpO_2_ remained at 100% throughout the intervention. While most previously reported cases were complicated by mask ventilation difficulty [4, 13], whereas our case presented as isolated difficult intubation with effective mask ventilation, a rare pattern that provides valuable reference data [14].

A missed diagnosis was the core reason for this accidental difficult airway [15]. Chest CT failed to detect the cyst, mainly because it does not routinely cover the epiglottic region and has insufficient resolution [16]. Moreover, the patient’s occasional snoring did not attract the attention of the anaesthesiologists. Snoring is an important early warning sign of upper airway stenosis. Airway-related examinations should be further improved for such patients. Lin [6] documented seven patients with epiglottic cysts who were diagnosed preoperatively, allowing the surgical plan to be determined in advance. Before intubation, the doctor evaluated the feasibility of mask ventilation under sedation; however, multiple intubation attempts were unsuccessful. A retrospective study [13] revealed undiagnosed epiglottic cysts during intubation in four patients who underwent oral surgery under general anaesthesia. Among these patients, two presented with mild exertional dyspnoea or snoring, whereas the remaining patients were asymptomatic. In accordance with the 2025 Difficult Airway Society guidelines [11], patients with classic risk factors (e.g., snoring, obesity, a short thick neck, or maxillofacial deformities) should undergo further airway evaluation, such as laryngoscopy or cervical imaging, to clarify upper airway anatomy. Our case highlights an important practical extension of this principle: even patients without obvious airway symptoms but with subtle warning signs, such as occasional snoring, should be considered for targeted preoperative assessment (e.g., laryngoscopy or thin-slice cervical CT). Our experience underscores the need for heightened awareness of occult airway pathologies, even in seemingly low-risk patients to avoid missed preoperative airway assessment. Advanced imaging-based predictive tools powered by artificial intelligence may further improve the early detection of subtle supraglottic lesions before anaesthesia induction [17].

Management of difficult airways is a critical aspect of anaesthesia practice. Adequate preoxygenation prior to induction is essential to prolong the safe apnoea duration and mitigate the risk of perioperative hypoxemia [18]. In this case, although mask ventilation remained effective, the anaesthesiologist avoided excessive positive pressure to prevent cyst rupture and aspiration. Simultaneously, the rapid activation of the difficult airway emergency protocol provided a vital safety buffer for subsequent airway establishment. Therefore, ensuring that tracheal intubation equipment is readily available when needed is essential. In cases of failed intubation, supraglottic airway devices can serve as effective alternatives [14].

Unexpected difficult airways caused by asymptomatic, giant epiglottic cysts involve multiple departments, including anaesthesiology, otolaryngology, and general surgery. Multidisciplinary collaboration is important for ensuring the safety of patients’ airways [12]. In this case, after the airway crisis occurred during the anaesthesia induction period, an otolaryngologist was immediately consulted to prepare for potential emergency tracheotomy, providing important support for airway management. The anaesthesia plan for the subsequent epiglottic cyst resection was jointly formulated by the departments of anaesthesiology and otolaryngology. This plan clarified the key points and emergency measures for airway management, ensuring the smooth progress of the surgery and anaesthesia.

### Limitations

This is a single-centre, single-patient case report; it presents clinical experiences that cannot be widely generalized. The speculation that the intact cyst wall maintained effective mask ventilation is based on clinical observation without pathological or imaging verification. Additionally, routine chest CT cannot effectively screen for epiglottic lesions, and the lack of targeted preoperative airway evaluation, such as flexible laryngoscopy, led to the missed diagnosis of this occult mass.

## Conclusion

Asymptomatic giant epiglottic cysts are easily overlooked preoperatively and can induce life-threatening unexpected difficult airways during anaesthesia induction. This case indicates that isolated difficult intubation with effective mask ventilation is a rare but clinically important presentation and that video laryngoscopy combined with a two-person cooperative manoeuvre contributes to the safe and rapid airway establishment. Ultimately, a heightened clinical awareness of subtle warning signs (e.g., snoring) and targeted preoperative airway evaluation are critical for reducing the risk of an unanticipated difficult airway and improving perioperative safety.

## Supplementary Information

Supplementary Material 1.

Supplementary Material 2.

## Data Availability

All data generated or analyzed during this study are included in this published article. The datasets used and/or analyzed during the current study are available from the corresponding author on reasonable request.

## References

[1] Alrashdan H, Alshugran K, Alshiyyab OM, et al. An Uncommon Epiglottic Cyst Presentation in an Adolescent: A Case Report and Literature Review[J]. Cureus. 2024;16(2):e54634. 10.7759/cureus.54634 PMC10959541 38524016

[2] Hou X, Huang N, Zhang X, et al. Epiglottic Cyst With Slurred Speech Misdiagnosed as Acute Cerebral Infarction: A Case Report. Neurologist[J]. 2023;28(1):39–41. 10.1097/NRL.0000000000000429 PMC9812411 35314586

[3] Uraguchi K, Otsuka Y, Marunaka H et al. Upper airway obstruction due to congenital epiglottic cyst: Report of two cases[J]. Congenit Anom (Kyoto) 2024,64(4):184–5. 10.1111/cga.12571 38716560

[4] Han S, Yang G. Asymptomatic giant epiglottic cyst causing an unexpectedly difficult airway: a case report[J]. BMC Anesthesiol. 2025;25(1):85. 10.1186/s12871-025-02948-9 PMC11837400 39972287

[5] Cai Y, Zhang L, Wang J, et al. Multidisciplinary management of extensive cervical lymphatic malformations and epiglottic cyst in a neonate: a case report and review of perioperative strategies[J]. Front Pediatr. 2025;13:1543250. 10.3389/fped.2025.1543250 PMC12450970 40989823

[6] Lin N, Li M, Shi S et al. Shikani™ seeing optical stylet-aided tracheal intubation in patients with a large epiglottic cyst[J]. Chin Med J (Engl) 2011,124(17):2795–8. 22040447

[7] Lee Y, Park HJ, Bae IH, et al. Resonance Characteristics in Epiglottic Cyst: Formant Frequency, Vowel Space Area, Vowel Articulatory Index, and Formant Centralization Ratio[J]. J Voice. 2024;38(2):273–8. 10.1016/j.jvoice.2021.09.008 34642071

[8] Varathan V, Mat Baki M, Kalimuthu S. A Case Report of an Unusual Presentation of Epiglottic Cyst Causing Airway Obstruction in an Adult[J]. Cureus. 2023;15(3):e35863. 10.7759/cureus.35863 PMC10078853 37033535

[9] Deng X, Diao M, Zhou J. A giant congenital epiglottic cyst in a neonate: a case report[J]. BMC Anesthesiol. 2025;25(1):107. 10.1186/s12871-025-02984-5 PMC11866621 40016678

[10] Cai H, Gao J. Low-Temperature Plasma Radiofrequency Ablation for Congenital Epiglottic Cysts[J]. Ear Nose Throat J. 2025;104(6):351–4. 10.1177/01455613241259368 38828889

[11] Ahmad I, El-Boghdadly K, Iliff H, et al. Difficult Airway Society 2025 guidelines for management of unanticipated difficult tracheal intubation in adults[J]. Br J Anaesth. 2026;136(1):283–307. 10.1016/j.bja.2025.10.006 41203471

[12] Manthirasalam D, Ramesh G. Airway Management Challenges in Three Cases of Vallecular Cysts[J]. Cureus. 2025;17(8):e90213. 10.7759/cureus.90213 PMC12439445 40964564

[13] Takaishi K, Otsuka R, Fujiwara SJ, et al. A retrospective case series of anesthetic patients with epiglottic cysts[J]. Anesth Prog. 2021;68(3):168–77. 10.2344/anpr-68-01-01 PMC8500314 34606575

[14] Martins MP, Ortenzi AV, Perin D, et al. Recommendations from the Brazilian Society of Anesthesiology for difficult airway management in adults[J]. Braz J Anesthesiol. 2024;74(1):447–447. 10.1016/j.bjane.2023.12.001 PMC10877351 38135152

[15] Kc AK, Shrestha BL. Multiple Epidermal Inclusion Cysts of Epiglottis[J]. Kathmandu Univ Med J (KUMJ) 2021,19(74):275–7. 34819451

[16] Xia M, Ma W, Zuo M, et al. Expert consensus on difficult airway assessment[J]. Hepatobiliary Surg Nutr. 2023;12(4):545–66. 10.21037/hbsn-23-46 PMC10432292 37600997

[17] De Rosa S, Bignami E, Bellini V, et al. The future of artificial intelligence using images and clinical assessment for difficult airway management[J]. Anesth Analg. 2025;140(2):317–25. 10.1213/ANE.0000000000006969 PMC11687942 38557728

[18] Crístian de Carvalho C, Iliff HA, Santos Neto JM, et al. Effectiveness of preoxygenation strategies: a systematic review and network meta-analysis[J]. Br J Anaesth. 2024;133(1):152–63. 10.1016/j.bja.2024.02.028 38599916

