# Supplementary material for: A case report: anesthetic management of a patient with unexpected difficult airway due to an asymptomatic giant epiglottic cyst
Source: BMC Anesthesiol. 2026 Aug 13;26:665. doi: 10.1186/s12871-026-04181-4 (PMC13749665; doi:10.1186/s12871-026-04181-4)
Supplement: Supplementary file 1 [file 12871_2026_4181_MOESM1_ESM.pdf]

宁波大学附属阳明医院（余姚市人民医院）

医学伦理委员会伦理审查批件

批件号 ：2026-02-005

**项目名称**	A Case Report: Anesthetic Management of a Patient with Unexpected Difficult Airway Due to an Asymptomatic Giant Epiglottic Cyst 病例报告：一例因无症状巨大会厌囊肿导致意外困难气道患者的麻醉管理		
**申办方/资助方**	宁波大学附属阳明医院（余姚市人民医院）		
**受理号**	S2026-028	**申请专业**	麻醉专业
**主要研究者**	汪啸骏	**承担科室**	麻醉科
**审查类别**	初始审查	**审查方式**	快速审查
**审查日期**	2026年02月27日	**审查地点**	主审委员办公室
**审查文件清单**	1. 临床研究初始审查申请；<br>2. 临床研究方案(1.0,2026-02-22)；<br>3. 研究者履历；<br>4. 利益声明；<br>5. 免除知情同意书申请表		
**审评意见**	无		
**审查决定**	委员会对该项目的审查决定为： ☑批准		
**主任/副主任委员签字**			
**签发日期**	2026年02月27日		
**医学伦理委员会**	宁波大学附属阳明医院（余姚市人民医院）医学伦理委员会盖章<br>地址：余姚市城东路800号宁大附属阳明医院（余姚市人民医院）科教中心二楼20[illegible]<br>电话：0571-62619288 联系人：[illegible]（伦理[illegible]）		
**批件有效期**	自本伦理审查委员会初始审查批准之日起一[illegible]本临床研究应在本院启动。逾期未启动的，本批件自行废止。		
**年度/定期跟踪审查**	审查频率为该研究批准之日起每 12 月一次，首次请于2027年02月26日 前 1 个月递交研究进展报告。<br>伦理审查委员会有根据实际进展情况改变跟踪审查频率的权利。		

扫描全能王 创建

**声明**	本伦理审查委员会的职责、人员组成、操作程序及记录遵循《涉及人的生物医学研究伦理审查办法》、《涉及人的生命科学和医学研究伦理审查办法》、《涉及人的健康相关研究国际伦理准则》、《赫尔辛基宣言》、GCP 和ICH-GCP 等国际伦理指南和国内相关法律法规。

注意事项：

1.请遵循我国相关法律、法规和规章中的伦理原则。

2.请遵循经本伦理审查委员会批准的临床研究方案、知情同意书、招募材料等开展本研究，保护研究参与者的健康与权利。对研究方案、知情同意书和招募材料等的任何修改，均须得到本伦理审查委员会审查同意后方可实施。

3.在本院发生的SAE/SUSAR 以及研发期间安全性更新报告须按照NMPA/GCP 最新要求及时递交本伦理审查委员会，国内外其它中心发生的 SAE/SUSAR 需定期汇总、评估后递交本伦理审查委员会。

4.根据报告情况，本伦理审查委员会有权对其评估做出新的决定。

5.自今日起，无论研究开始与否，请在跟踪审查日到期前1个月提交研究进展报告。

6.申办方应当向组长单位伦理审查委员会提交中心研究进展报告汇总；当出现任何可能显著影响研究进行或增加研究参与者危险的情况时，请申请人及时向本伦理审查委员会提交书面报告。

7.研究纳入了不符合纳入标准或符合排除标准的研究参与者，符合中止研究规定而未让研究参与者退出研究，给予错误治疗或剂量，给予方案禁止的合并用药等没有遵从方案开展研究的情况；或可能对研究参与者的权益或健康以及研究的科学性造成不良影响等违背 GCP 原则的情况，请申办方、监查员或研究者提交违背方案报告。

8.申请人暂停或提前终止临床研究，请及时提交暂停或终止研究报告。

9.完成临床研究，请申请人提交结题报告。

10.采集、保藏、利用、对外提供我国人类遗传资源，应当遵守中华人民共和国人类遗传资源管理条例。

11.凡经本伦理审查委员会批准的研究项目在实施前，申请人应按相关规定在国家卫健委、药审中心等的临床研究登记备案信息系统平台登记研究项目相关信息。

扫描全能王 创建
