# Supplementary material for: A case report: anesthetic management of a patient with unexpected difficult airway due to an asymptomatic giant epiglottic cyst
Source: BMC Anesthesiol. 2026 Aug 13;26:665. doi: 10.1186/s12871-026-04181-4 (PMC13749665; doi:10.1186/s12871-026-04181-4)
Supplement: Supplementary file 2 [file 12871_2026_4181_MOESM2_ESM.pdf]

临床研究知情同意书

知情告知页

项目名称：A Case Report: Anesthetic Management of a Patient with Unexpected Difficult Airway Due to an Asymptomatic Giant Epiglottic Cyst

病例报告：一例因无症状巨大会厌囊肿导致意外困难气道患者的麻醉管理

主要研究者：汪啸骏 麻醉科主治医师

研究机构：宁波大学附属阳明医院（余姚市人民医院）三级乙等医院

尊敬的郑小兰女士：

我们将邀请您参加本项研究，在您决定是否参加这项研究之前，请尽可能仔细阅读以下内容，它可以帮助您了解该项临床治疗研究以及为何要进行这项研究，研究的程序和期限，参加研究后可能给您带来的益处、不适和风险。如果您愿意，您也可以和您的亲属、朋友一起讨论，或者请您的医生给予解释，帮助您做出决定是否参加此项临床研究。如有任何疑问请您向负责该项研究的医生提出。

一、研究背景及目的

研究背景：

会厌囊肿属于非癌性病变，其病因主要与黏液腺分泌通道阻塞及分泌物积聚有关。巨大会厌囊肿初期通常无明显症状，但随着逐渐增大可导致呼吸困难、咽部异物感、急性会厌炎等。无症状会厌囊肿患者在麻醉相关急症中可能面临气道管理困难，可能导致全身麻醉快速诱导期间通气不良或插管困难，极端情况下甚至需要行气管切开术。

研究目的：

本研究的目的是探讨麻醉诱导后无症状性巨大会厌囊肿患者意外困难气道的识别与处理策略，回顾并梳理相关意外困难气道患者麻醉管理方案，本研究通过病例分析和文献复习，旨在提高对本病的认识与识别，为此类患者的麻醉处理提供借鉴。

二、哪些人适宜参加研究

年龄 18~80 周岁，男女不限，无症状巨大会厌囊肿导致意外困难气道病例

三、哪些人不宜参加研究

年龄<18 或>80 周岁，不符合无症状巨大会厌囊肿导致意外困难气道的病例

四、研究过程及研究时限

本次研究开展的起止日期为：2025年7月-2026年2月；大概有1位研究参与者参加研究。

如果您自愿参加本项研究，并签署本知情同意书，研究人员将对您进行入组前的筛查，如果您符合入组条件，将被纳入本项研究，我们将收集您的完整病史资料(包括但不限于既往史、家族史和现病史)、详细的治疗记录(涵盖手术方案、完整的麻醉记录单)、多模态影像学数据以及实验室检验结果（包括但不限于电子喉镜检查报告）。

本研究采集的所有数据仅用于本项研究，检测后将按照研究中心规定销毁。检测结果可能有助于更好地了解您的疾病并提高整体诊疗水平。

五、风险和不便

本次研究的主要不良事件（风险）

（1）本研究的主要不良事件

您的个人信息泄露。

（2）本研究的主要不良事件的处理：

若发生信息泄露等不良事件，研究者将立即启动应急预案，第一时间采取有效措施控制风险、防止信息进一步扩散，并及时上报医院伦理委员会及相关管理部门，评估泄露范围与严重程度，按规定进行后续处置与整改，切实保护您的隐私与合法权益。

（3）严重不良事件的记录和报告

研究期间所有严重不良事件，无论是否与研究相关，应当立即对研究参与者采取适当的治疗措施，同时填写“严重不良事件报告表”，在24小时内报告余姚市人民医院医学伦理委员会。

六、参加研究的受益

研究参与者没有收益，社会将可能从本项研究中收获此类患者的真实数据，并指导之后同类情况下出现困难气道后的识别与处理。

七、参加研究是否可获得补偿

您参加本项研究将不获得任何经济补偿。

八、 参加研究是否需要承担费用

本研究不会额外增加您的经济负担。

九、发生损害的赔偿问题

如果您发生了与本研究有关的损害，研究员将按国家有关法律法规的规定进行赔偿和补偿。

十、隐私保护

如果您决定参加本项研究，您参加研究及在研究中的个人资料均属保密。您的身份信息将不会透露给研究团队以外的成员，除非获得您的许可。所有的研究团队成员都被要求对您的身份保密。

为确保研究按照规定进行，必要时，宁波大学附属阳明医院（余姚市人民医院）医学伦理委员会、政府管理部门出于监管的目的可以查阅含有您个人信息的原始医疗记录和您已署名的知情同意书。这些核查人员都承担了研究参与者信息保密责任。

如果这项研究被发表在研究期刊上，您的姓名和其他可识别您身份的信息都将删除，以研究参与者编号来替代。您的身份不会被披露，未经授权的个人在理论上也不可能识别出您的身份。我们将在法律允许的范围内，尽一切努力保护您个人资料的隐私。

十一、自愿选择参加研究和中途退出研究

是否参加研究完全取决于您的自愿，您可以和您的家人或者朋友讨论后再做出决定。在您做出参加研究的决定前，请尽可能向研究人员询问有关问题，直至您对本知情同意书的内容完全理解。

您可以随时退出研究，且无需提供任何理由。这不会影响您和研究人员之间的关系，也不会造成您的医疗或其他方面利益的损失。我们将保留并使用截至您退出时采集的研究数据。

如果研究不再符合您的最佳利益或者因为其他科学或安全性原因，研究人员或研究发起者也有权利决定您退出研究。如果研究人员终止您继续参与本研究，他/她会跟您讨论主要原因。

十二、关于研究的咨询

研究人员将给您留下他/她的电话号码以便您能与他/她联系。您可以在任何时间提出有关本项研究的任何问题。如果在研究过程中有任何重要的新信息，包括但不限于不良事件和重大发现，可能影响您继续参加研究的意愿时，研究人员将会及时通知您。您会被要求重新签一份知情同意书来记录您获得的更新的消息以及您愿意继续参与本项研究。

如果您对研究过程、研究中的权利等任何方面有疑问，或者您认为自己受到了研究相关的损害，请联系研究人员 汪啸骏 ，联系电话 13626800025。

如果您对参与本项目的权利存在疑问或有任何不满，请联系：

1. 医学伦理委员会名称：宁波大学附属阳明医院（余姚市人民医院）医学伦理委员会，座机：0574-62619288；
2. 医学伦理委员会办公室地址：余姚市城东路 800 号，宁波大学附属阳明医院（余姚市人民医院）科教中心二楼 204 室

知情同意书签字页

项目名称：A Case Report: Anesthetic Management of a Patient with Unexpected Difficult Airway Due to an Asymptomatic Giant Epiglottic Cyst

病例报告：一例因无症状巨大会厌囊肿导致意外困难气道患者的麻醉管理

主要研究者：汪啸骏 麻醉科主治医师

研究机构：宁波大学附属阳明医院（余姚市人民医院）三级乙等医院

研究参与者/或监护人同意声明：

我已经阅读了上述有关本研究的介绍，而且有机会就此项研究与医生讨论并提出问题。我提出的所有问题都得到了满意的答复。

我知道参加本研究可能产生的风险和受益。我知晓参加研究是自愿的。我确认已有充足时间对此进行考虑，而且明白：

1. 我可以随时向医生咨询更多的信息。
2. 我可以随时退出本研究，而不会受到歧视或报复，医疗待遇与受益不会受到影响。

我将获得一份经过签名并注明日期的知情同意书副本。我同意卫生管理监督部门、伦理委员会或专业学术委员会查阅我的研究资料。

最后，我决定同意参加本项研究。

研究参与者姓名（正楷书写）郑小兰；

研究参与者签名：郑小兰　　　　签字日期2025年7月10日

身份证号码：|4|1|2|8|2|8|1|9|6|5|0|4|0|8|0|9|4|4|

联系方式：（手机）13884473400（固定电话）0574-62639345

监护人（适用时）

当研究参与者无知情同意能力、或不能充分地给予知情同意（如未成年人，或患严重痴呆者），以及紧急情况时，应向（或同时向）其监护人作知情同意，获得其合法代表的同意。当研究参与者能做出同意参加研究决定时，还必须征得其本人同意]

研究参与者监护人姓名（正楷书写）______________；　（与其关系）__________；

研究参与者监护人签名：_____________　　　签字日期_____年___月___日

身份证号码:|__|__|__|__|__|__|__|__|__|__|__|__|__|__|__|__|__|__|

联系方式:（手机）______________;（固定电话）______________;

见证人（适用时）

研究参与者或其监护人无足够的阅读理解能力时，须详细向研究参与者本人或其监护人作知情同意，并由见证此过程的人员签名确认，如研究参与者或其监护人能签名，也需要同时签名]

本人证明：研究人员已将本知情同意书中的信息准确地向研究参与者或其监护人进行了解释，且研究参与者或其监护人已充分理解这些信息。**本人同时证明：**我作为见证人在此签名证实，该研究参与者或其监护人在我在场的情况下，完全自愿同意参加这项研究。

见证人姓名（正楷书写）：__________ 签字日期_____年___月___日

身份证号码:|__|__|__|__|__|__|__|__|__|__|__|__|__|__|__|__|__|__|

联系方式:（手机）______________;（固定电话）______________;

研究者声明

我确认已向患者解释了本项研究的详细情况，包括其权力以及可能的受益和风险，并给其一份签署过的知情同意书副本。

医生（研究者）姓名 王晓?（签名）；签字日期 2025 年 7 月 10 日

联系方式:（手机） 13626800025;（固定电话） 0574-62619577;（其他方式）wxj3037377@163.com.

（此页为研究参与者知情同意书的必要部分，每一份“研究参与者知情同意书”必须有研究参与者或法定代理人及研究医生的签字和日期，方为有效。）

如果您对参与本项目的权利存在疑问或有任何不满，请联系：

1. 医学伦理委员会名称：宁波大学附属阳明医院（余姚市人民医院）医学伦理委员会，座机：0574-62619288;
2. 医学伦理委员会办公室地址：余姚市城东路 800 号，宁波大学附属阳明医院（余姚市人民医院）科教中心二楼 204 室
